# Antitumor activities of Aspiletrein A, a steroidal saponin from *Aspidistra letreae,* on non-small cell lung cancer cells

**DOI:** 10.1186/s12906-021-03262-w

**Published:** 2021-03-09

**Authors:** Hien Minh Nguyen, Hoai Thi Nguyen, Suthasinee Seephan, Hang Bich Do, Huy Truong Nguyen, Duc Viet Ho, Varisa Pongrakhananon

**Affiliations:** 1 Faculty of Pharmacy, Ton Duc Thang University, Ho Chi Minh City, Vietnam; 2 Faculty of Pharmacy, Hue University of Medicine and Pharmacy, Hue University, Hue City, Vietnam; 3 Pharmaceutical Sciences and Technology Graduate Program, Faculty of Pharmaceutical Sciences, Chulalongkorn University, Bangkok, 10330 Thailand; 4 Department of Pharmacology and Physiology, Faculty of Pharmaceutical Sciences, Chulalongkorn University, Bangkok, 10330 Thailand; 5 Preclinical Toxicity and Efficacy Assessment of Medicines and Chemicals Research Cluster, Chulalongkorn University, Bangkok, 10330 Thailand

**Keywords:** *Aspidistra letreae*, Aspiletrein A, Anti-proliferation, Anti-migration, Anti-invasion, Lung cancer cells

## Abstract

**Background:**

Lung cancer is one of the leading causes of death worldwide due to its strong proliferative and metastatic capabilities. The suppression of these aggressive behaviors is of interest in anticancer drug research and discovery. In recent years, many plants have been explored in order to discover new bioactive secondary metabolites to treat cancers or enhance treatment efficiency. Aspiletrein A (AA) is a steroidal saponin isolated from the whole endemic species *Aspidistra letreae* in Vietnam. Previously, elucidation of the structure of AA and screening of its cytotoxic activity against several cancer cell lines were reported. However, the antitumor activities and mechanisms of action have not yet been elucidated. In this study, we demonstrated the anti-proliferative, anti-migrative and anti-invasive effects of AA on H460, H23 and A549 human lung cancer cells.

**Methods:**

MTT, wound healing and Transwell invasion assays were used to evaluate the anti-proliferation, anti-migration and anti-invasion effects of AA, respectively. Moreover, the inhibitory effect of AA on the activity of protein kinase B (Akt), a central mediator of cancer properties, and apoptotic regulators in the Bcl-2 family proteins were investigated by Western blotting.

**Results:**

AA exhibits antimetastatic effects in human lung cancer cells through the inhibition of the pAkt/Akt signaling pathway, which in turn resulted in a significant inhibitory effect of AA on the migration and invasion of the examined lung cancer cells.

**Conclusions:**

Aspiletrein A may be a potent inhibitor of protein kinase B (Akt). Hence, AA could be further explored as a potential antimetastatic lead compound.

**Supplementary Information:**

The online version contains supplementary material available at 10.1186/s12906-021-03262-w.

## Background

The incidence and mortality rates of cancer are sharply increasing every year. Globally, the burden of cancer increased to approximately 18.1 million new cases and 9.6 million deaths in 2018 [1, 2]. Lung cancer is one of the most commonly occurring cancers and common causes of death. It is divided into two types, small-cell lung carcinoma (SCLC) and non-small cell lung carcinoma (NSCLC) [3–6]. NSCLC accounts for the majority of lung cancer cases and exhibits a strong capacity for metastasis. Most patients are in an advanced stage at first diagnosis, and the survival rate of metastatic lung cancer patients is extremely low, at only 5% [7]. Metastasis, one of the hallmark characteristics of cancer, comprises multiple processes: cancer detachment from primary organs, migration and invasion through surrounding tissues, intravasation into systemic circulation, extravasation into secondary sites and secondary tumor establishment [8–10]. Several signaling pathways governing cancer aggressiveness, including protein kinase B (Akt), have been identified. Akt is a serine- and threonine-specific protein kinase that plays an important role as an oncogenic promotor [11]. An increase in active Akt or its phosphorylation (pAkt) enhances the cell growth, cell survival and metastasis abilities of lung cancer, and the attenuation of Akt function has become a promising strategy for the research and development of anti-lung cancer therapies [12]. Although current therapeutic drugs, including cisplatin, paclitaxel and etoposide, are recommended and efficient for lung cancer treatment, cancer metastasis and chemotherapeutic resistance often occur [13, 14]. Therefore, the discovery of novel anticancer agents for lung cancer is urgently required.

**Fig. 1:**
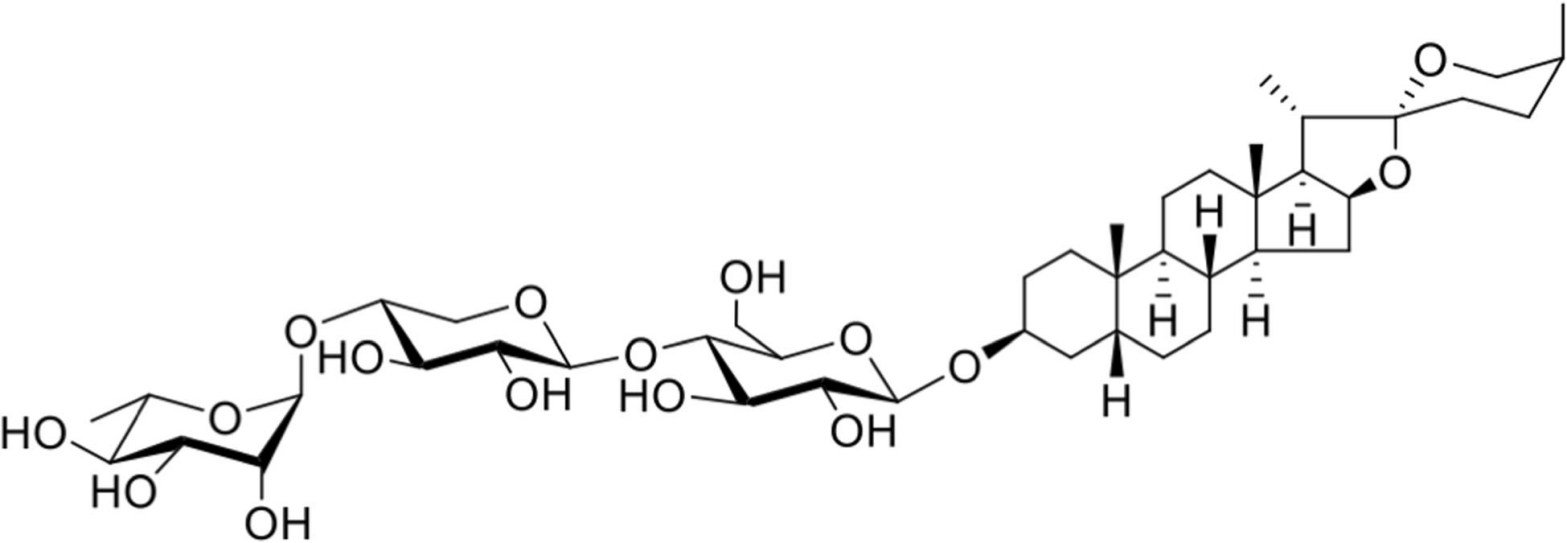
Chemical structure of Aspiletrein A from *Aspidistra letreae*

*Aspidistra letreae* Aver., Tillich and T.A. Le is a new species in the *Aspidistra* genus that was discovered in 2016 [15]. Traditionally, *Aspidistra* has been widely used in the forms of tonics, expectorants, diuretics, and treatments for fractures, congestion and snakebites [16–18]. *Aspidistra* contains rich chemical components, including saponins, lectins, and homoisoflavones. Additionally, this genus is a potential source of secondary metabolites with a broad spectrum of biological activities, including antifungal [19], antitumor [20], antibacterial [21] and cytotoxic activities [22–24]. Previously, phytochemical investigations of active steroidal saponins led to their isolation and characterization, and aspiletrein A (AA) (Fig. 1) showed the greatest cytotoxicity against five human cancer cell lines, including lung adenocarcinoma (LU-1), cervical carcinoma (HeLa), breast adenocarcinoma (MDA-MB-231), liver hepatocellular carcinoma (HepG2) and gastric adenocarcinoma (MKN-7) cell lines, with IC_50_ values less than 12.5 μM [25]. In the present study, we continued investigating the in vitro anticancer activities, particularly the antiproliferation, antimigration and anti-invasion activities, of AA in H460, H23 and A549 lung cancer cells. We found that AA suppressed the migration and invasion of these cancer cell lines through Akt signaling. The results of this study might provide scientific information on AA for future anticancer research and development.

## Methods

### Test compound and reagents

Aspiletrein A (AA), a dry powder, was isolated and characterized as previously described [25]. The structure was determined by NMR analysis on a Bruker Avance 500 spectrometer (Bruker, MA, USA), and the HRESIMS was recorded on an Agilent 6545 accurate-mass spectrometer (Agilent, CA, USA). AA: White amorphous powder [α]^25^_D_ − 92.0 (*c* 0.1, MeOH); IR (KBr) *ν*_max_ (cm^− 1^): 3422, 2928, 1632, 1454, 1379, 1344, 1221, 1165, 1049, 986, 918, 847, 810; ^1^H NMR (500 MHz, pyridine-*d*_5_) and ^13^C NMR (125 MHz, pyridine-*d*_5_), HRESIMS *m/z* 891.4522 [M + Cl]^−^ (calcd. For C_44_H_72_O_16_Cl, 891.4509) [25]. AA was dissolved in DMSO to yield a stock solution and further diluted in complete medium to the desired concentrations before use. The control samples in the experiments were incubated with 0.1% DMSO in the culture medium. The final concentration of DMSO was less than 0.1%, which showed no toxicity.

Hoechst 33342, propidium iodide, 3-(4,5-di-methylthiazol-2-yl)-2,5-diphenyltetrazolium bromide (MTT) and dimethyl sulfoxide (DMSO) were obtained from Sigma Chemical, Inc. (St. Louis, MO, USA). Rabbit anti-phosphorylated Akt (S473), rabbit anti-Akt and HRP-linked anti-rabbit IgG were purchased from Cell Signaling Technology (Beverly, MA, USA).

### Cell culture

Human lung cancer H460 (HTB-177), H23 (CRL-5800) and A549 (CCL-185) cells were obtained from ATCC (Rockville, MD, USA). H460 and H23 cells were cultured in RPMI-1640, and A549 cells were cultured in DMEM. All the cell cultures were supplemented with 10% fetal bovine serum (FBS), 2 mM L-glutamine and 100 units/mL penicillin/streptomycin and incubated in 5% CO_2_ at 37 °C. The media and supplements were obtained from Thermo Fisher Scientific (Waltham, MA, USA).

### Cytotoxicity and cell proliferation assays

Cytotoxicity was assessed by MTT assay, as previously reported [26]. Briefly, 10^4^ cells/well were seeded into 96-well plates and allowed to attach at 37 °C in 5% CO_2_ overnight. Then, AA (0–50 μM) was added and incubated for 24 h. The cells were washed with PBS, and 100 μL of 500 μg/mL MTT solution was added to each well. After incubation for 3 h, the formazan crystals were solubilized with 100 μL DMSO. The optical absorption of the formazan product was measured at 570 nm using a microplate reader (Perkin Elmer VICTOR3/Wallac1420), and DMSO was used as the blank. Cell viability was calculated from the mean values of the data as a percentage of the control. The IC_50_ (50% inhibitory concentration) value was determined by Prism 8 (GraphPad Software, CA, USA).

For cell proliferation assessment, a low number of cells (2 × 10^3^ cells/well) were seeded into 96-well plates and allowed to attach at 37 °C in 5% CO_2_ overnight. The cells were treated with a noncytotoxic concentration of AA for 48 h, which included the doubling time of the cells (approximately 24 h), and cell growth was evaluated by MTT assay, as described above. The data are presented as a percentage of growth in the treated cells compared to that in the control cells.

### Cell migration assay

Cell migration was determined using a wound healing assay [26, 27]. Briefly, a total of 2 × 10^5^ cells/well were seeded into 24-well plates and allowed to attach overnight. After a cell monolayer was formed, a wound space was generated using a micropipette tip. The cells were then washed with PBS and treated with various concentrations of AA in low-serum medium (1% FBS) for 48 h. The wound spaces were imaged and quantified as a relative migration level compared to that in the control cells.

### Cell invasion assay

A total of 3 × 10^4^ cells/well in low-serum medium (1% FBS) were seeded in Matrigel-coated Transwell®-Clear Inserts with polyester membrane, and complete medium (10% FBS) was added to the lower chamber. The cells were incubated with various concentrations of AA for 24 h. The cells on the upper side of the membrane were removed with cotton swabs, and the cells attached to the underside of the membrane were fixed with cold methanol at − 20 °C for 5 min, stained with 10 μg/mL DAPI for 15 min and visualized by fluorescence microscopy (Nikon Inverted Microscope Eclipse Ti-U Ti-U/B, NY, USA). Five random fields were captured and analyzed. The data are presented as the percentage of invaded cells in the treated cells compared to that in the control cells.

### Western blot analysis

A total of 7.5 × 10^5^ cells/dish were seeded into 60-mm cell culture dishes overnight. The cells were treated with various concentrations of AA for 24 h and lysed in lysis buffer (containing 20 mM Tris-HCl pH 7.5, 1 mM MgCl_2_, 150 mM NaCl, 20 mM NaF, 0.5% sodium deoxycholate, 1% nonidet-40, 0.1 mM phenylmethylsulfonyl fluoride, and protease inhibitor cocktail (Corning, NY, USA)) on ice for 40 min. The supernatants were collected by centrifugation at 12,000 xg at 4 °C for 15 min. The protein content was quantified by a BSA protein assay kit (Thermo Fisher Scientific, MA, USA). Protein lysate was boiled in 6X sampling at 95 °C for 5 min. Aliquots (60 μg) of protein were separated in SDS-polyacrylamide gels and electrotransferred onto polyvinyl difluoride (PVDF) membranes. The membranes were blocked in 5% skim milk in Tris-buffered saline with 0.75% Tween 20 (TBS-T) for 1 h at room temperature, incubated with rabbit primary antibodies against pAkt (S437) or Akt at 4 °C overnight, and incubated with secondary antibodies at room temperature for 2 h. An anti-glyceraldehyde-3-phosphate dehydrogenase (GAPDH) antibody was used as a loading control. The proteins were visualized by an enhanced chemiluminescence system using Immobilon Western chemiluminescent HRP substrate (Millipore, MA, USA) and quantified by ImageJ software. The full-length blots are presented in Supplementary figures.

### Statistical analysis

The data are presented as the mean ± SEM obtained from three independent experiments and analyzed by Prism 8 (GraphPad Software, CA, USA). Student’s *T-test* was used to determine statistical significance between two groups, and one-way ANOVA with Tukey’s multiple comparison test was used for multiple groups. *P*-values less than 0.05 were considered significantly different.

## Results

### Cytotoxicity of AA on H460, H23 and A549 human lung cancer cells

**Fig. 2:**
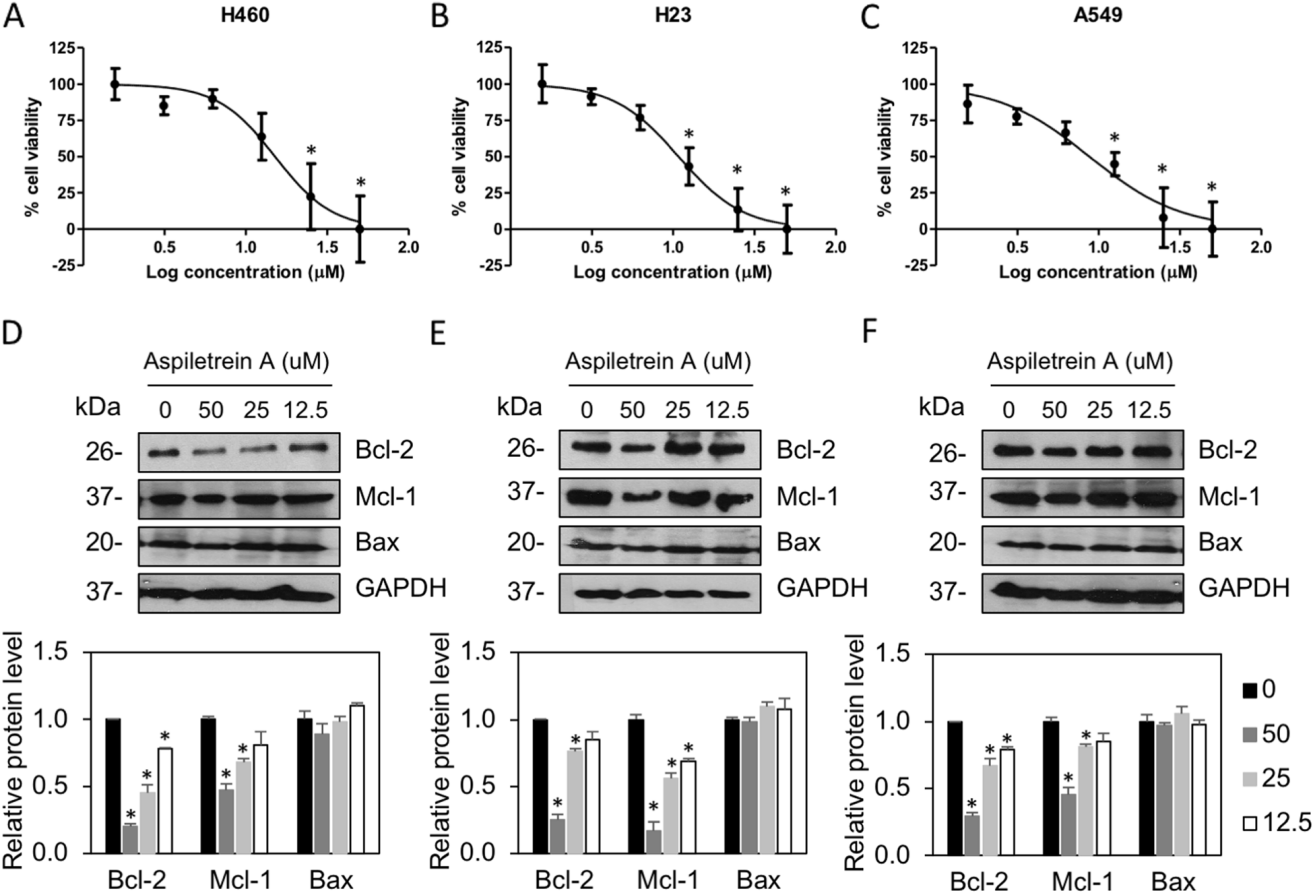
The effects of AA on the viability of human lung cancer cells. H460 (**a**), H23 (**b**), and A549 (**c**) cells were treated with AA (0, 1.5625, 3.125, 6.25, 12.5, 25.0 and 50 μM) for 24 h. Cell survival was evaluated by MTT assay. The plots present the percentage of cell viability ± SEM from three independent experiments (*n* = 3). *, *p* < 0.05 vs nontreated cells. H460 (**d**), H23 (**e**) and A549 (**f**) cells were treated with AA (0–50 μM) for 24 h. Anti-apoptotic Bcl-2 and Mcl-1 and pro-apoptotic Bax were examined by Western blot assay. Blots were reprobed with anti-GAPDH antibodies to confirm equal loading. The protein levels were quantified and normalized to the glyceraldehyde-3-phosphate dehydrogenase (GAPDH) level. The data are the mean ± SEM from three independent experiments (n = 3). *, *p* < 0.05 vs nontreated cells

A previous study demonstrated that AA exhibited the strongest cytotoxicity among isolated saponins and exerted potent effects against the LU-1 cell line, with an IC_50_ value of 9.94 ± 2.15 μM [25]. The cytotoxic effect was extensively investigated in H460, H23 and A549 non-small cell lung cancer cells by MTT assay. The results showed that treatment with AA caused a dose-dependent decrease in cell survival (Fig. 2). AA exhibited significant cytotoxicity against the H460, H23 and A549 cell lines, especially at higher doses (≥ 25 μM), with IC_50_ values of 15.13 ± 4.86, 10.10 ± 2.93 and 8.78 ± 3.08 μM, respectively (Table 1). AA exhibited less cytotoxicity against normal bronchial epithelial cells, with IC_50_ values of 26.09 ± 4.08 μM. The selectivity index calculated as the IC_50_ of normal cells/IC_50_ of cancer cells, demonstrated that AA has a selectivity index in the range of 1.7–2.9, indicating that this compound preferentially induced cytotoxicity in lung cancer. Generally, the present results are in good agreement with our previous study [25].

**Table 1:** Cytotoxicity of AA against human lung cancer and human bronchial epithelial cell lines

Lung cancer cell lines	^a^IC_**50**_ (μM ± SD)	Bronchial epithelial cell line	^a^IC_**50**_ (μM ± SD)	^b^SI
H460	15.13 ± 4.86	BEAS-2B	23.09 ± 4.08	1.72
H23	10.10 ± 2.93			2.58
A549	8.78 ± 3.08			2.97

^a^Concentration that inhibits cell viability by 50%. ^b^Selectivity index = IC_50_ of normal cells/IC_50_ of cancer cells [28]

Furthermore, the effect of AA on apoptotic regulatory proteins was examined. Western blot analysis revealed that antiapoptotic Bcl-2 and Mcl-1 were gradually decreased in response to AA treatment, whereas no change was observed in proapoptotic Bax. These data indicated that AA-induced lung cancer cell death might be caused by downregulation of the Mcl-1 and Bcl-2 levels.

### Anti-proliferative effect of AA on H460, H23 and A549 human lung cancer cells

**Fig. 3:**
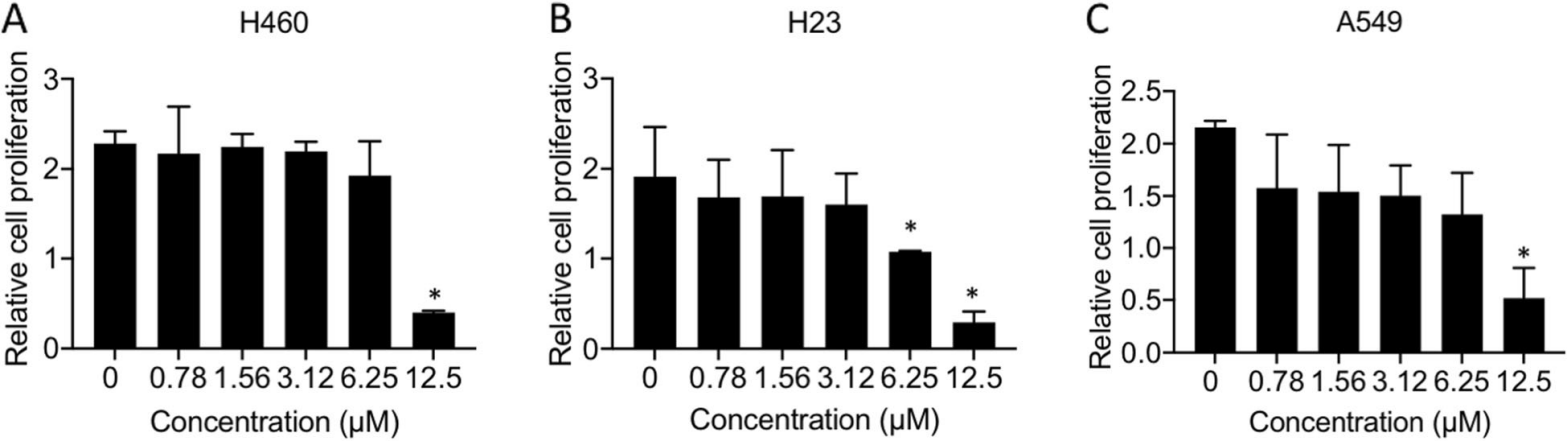
Anti-proliferative effect of AA on human lung cancer cells. H460 (**a**), H23 (**b**) and A549 (**c**) cells were treated with AA (0–12.5 μM) for 48 h. Cell growth was evaluated by MTT assay. The plots present the relative cell proliferation ± SEM from three independent experiments (n = 3). *, *p* < 0.05 vs nontreated cells

Uncontrolled cell growth is recognized as an important feature of cancer [29]. To determine whether AA was able to suppress cancer cell growth, the antiproliferative effect of AA was examined. Lung cancer cells were treated with low doses (0–12.5 μM) of AA for 48 h, and cell proliferation was evaluated by MTT assay. The results showed that AA concentrations lower than 6.25 μM did not show any significant effect on the proliferation of H460 and A549 cells and that 3.13 μM AA did not show any significant effect on the proliferation of H23 cells up to 72 h (Fig. 3). However, at a higher dose of AA (12.5 μM), cell proliferation was dramatically decreased at both 48 h and 72 h. The reduction in the cell numbers after treatment with AA was mainly caused by the cytotoxicity of AA rather than its actual effect on cell growth.

### Anti-migration and anti-invasion effects of AA on H460, H23 and A549 human lung cancer cells

**Fig. 4:**
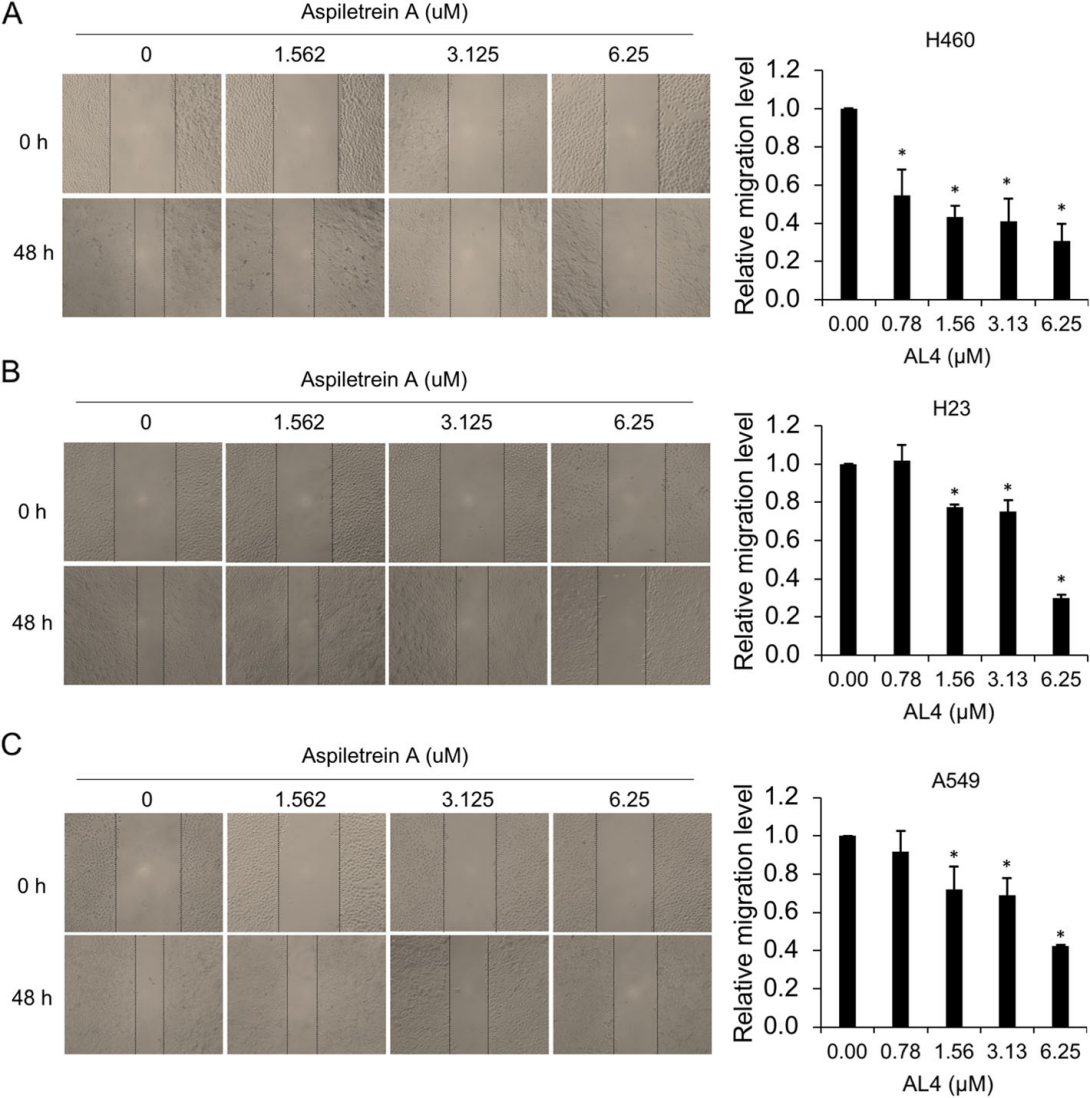
Antimigration effects of AA on human lung cancer cells. H460 (**a**), H23 (**b**) and A549 (**c**) cells were treated with AA (0–6.25 μM) for 48 h. Cell migration was evaluated by wound healing assay. The wound area was calculated and is presented as a relative migration level compared to that in the control group. The data are the mean ± SEM from three independent experiments (n = 3). *, *p* < 0.05 vs nontreated cells

Since cell migration and invasion are required for cancer metastasis, the negative effect of AA on these metastatic phenotypes was investigated. A wound healing assay was performed in cells treated with nontoxic doses (0–6.25 μM) of AA in low-serum medium to avoid the interference of antiproliferative activity. The results demonstrated that the amount of lung cancer cell migration to close the wound space was attenuated by AA, and the wound area that remained in the AA-treated group, especially the group treated with a high dose (6.25 μM), was obviously greater than that in the control group. AA at doses of 1.56, 3.13 and 6.25 μM notably inhibited the migratory behavior of the H460, H23 and A549 cells compared to that of the control cells (Fig. 4). In particular, the antimigration effects of AA on H460 cells was initially observed at a low dose of 0.78 μM. The mobility of H460 cells decreased to 55, 43, 40 and 34% in response to 0.78, 1.56, 3.13 and 6.25 μM AA, respectively. However, cell migration was decreased to approximately 75, 73, and 36% in H23 cells treated with 1.56, 3.13 and 6.25 μM AA, respectively. The pattern of AA activity on A549 cells was similar to that of its activity on H23 cells. These results indicated that AA possessed the ability to inhibit lung cancer cell migration.

**Fig. 5:**
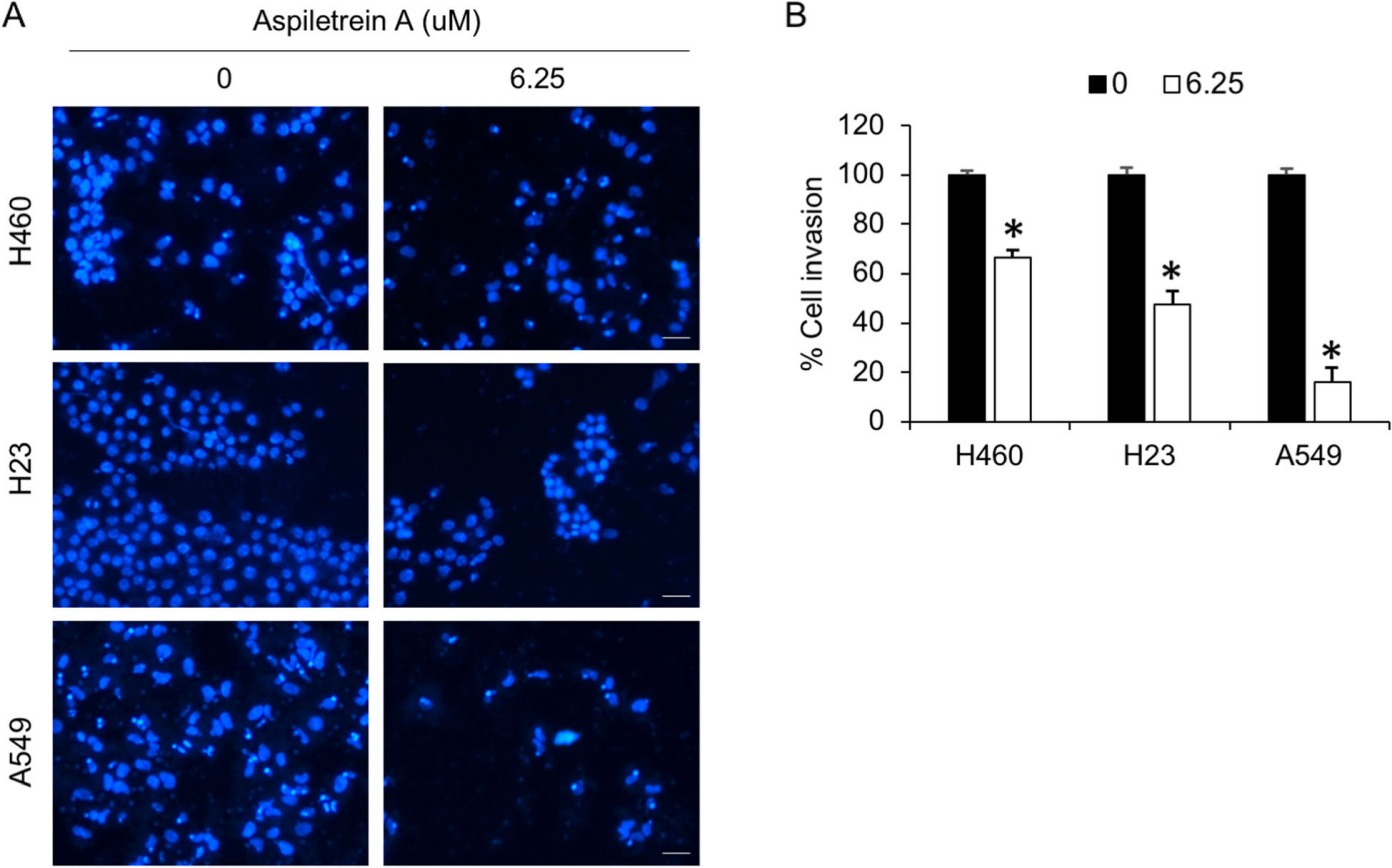
Anti-invasion effects of AA on human lung cancer cells. H460, H23 and A549 cells were treated with AA (6.25 μM) for 24 h. Cell invasion was evaluated by Transwell invasion assay. **a** The invaded cells were imaged in at least five random fields and calculated as a percentage of those in the control group. **b** The data are the mean ± SEM from three independent experiments (n = 3). *, *p* < 0.05 vs nontreated cells. Scale bar = 20 μm

Furthermore, the effect of AA on NSCLC invasion was evaluated by Transwell invasion assay. Compound AA at a concentration of 6.25 μM strongly attenuated the invasive capabilities of all the cells at 24 h, and the most significant result was observed in A549 cells (Fig. 5). Fluorescence microscopy images revealed a reduction in the number of invaded cells in the treated samples compared to the control samples. Taken together, these results indicated the potent antimetastatic activity of AA in NSCLC.

### AA-mediated suppression of human lung cancer cell migration and invasion via Akt signaling

**Fig. 6:**
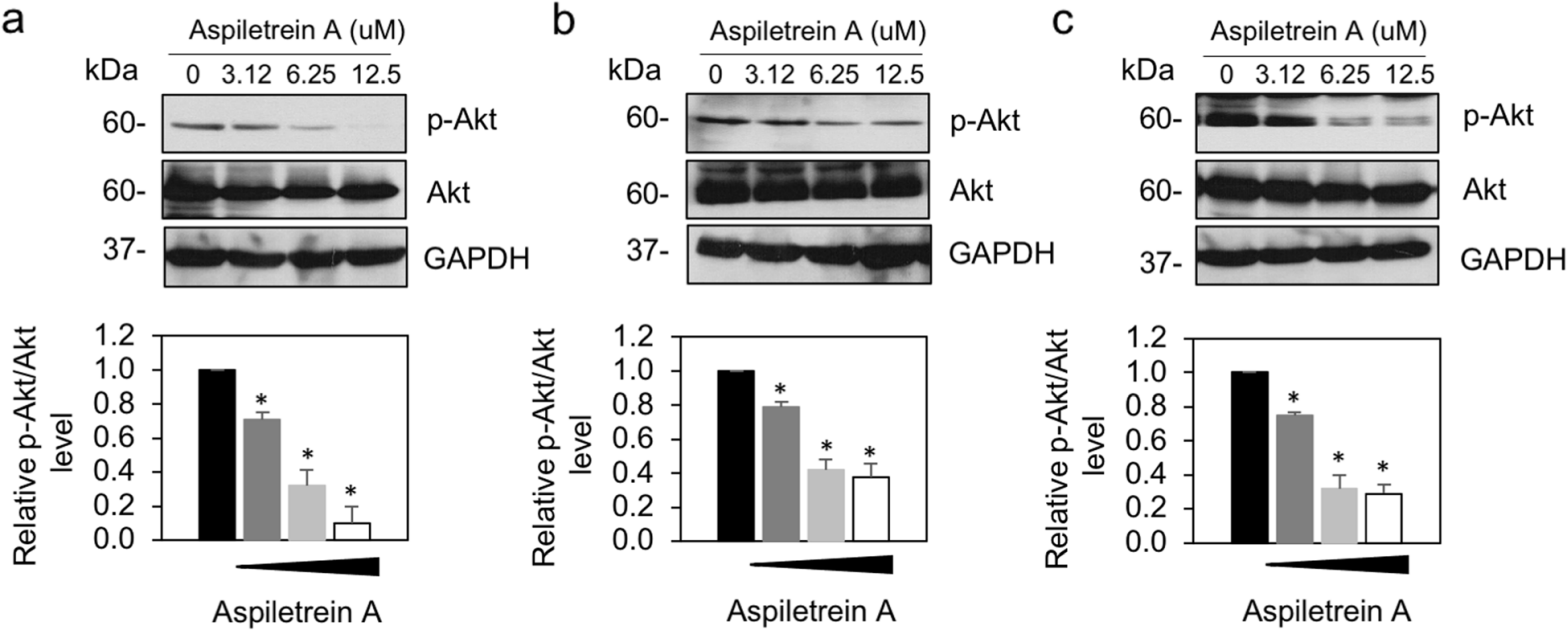
The effect of AA on Akt signaling in human lung cancer cells. H460 (**a**), H23 (**b**) and A549 (**c**) cells were treated with AA (0–12.5 μM) for 24 h. Phosphorylated Akt (pAkt^S473^) and total Akt were examined by Western blot assay. Blots were reprobed with anti-GAPDH antibodies to confirm equal loading. The level of pAkt to Akt was quantified and normalized to the glyceraldehyde-3-phosphate dehydrogenase (GAPDH) level. The data are the mean ± SEM from three independent experiments (n = 3). *, *p* < 0.05 vs nontreated cells

Akt signaling has been reported to govern sporadic cancer [11], and phosphorylated Akt (pAkt^S473^) and total Akt were investigated. Western blot analysis revealed that the pAkt level was markedly downregulated in all the examined cells in a dose-dependent manner (Fig. 6). The pAkt/total Akt ratio observed in H460, H23 and A549 cells was clearly reduced by 0.32-, 0.42- and 0.35-fold in response to 62.5 μM AA and by 0.11-, 0.38- and 0.29-fold in response to 12.5 μM AA, respectively, whereas the total Akt levels were unchanged. These results indicated that pAkt/Akt participates in the antimetastatic effect of AA in human lung cancer cells.

## Discussion

Non-small cell lung carcinoma is the most common type of lung cancer, and metastasis and apoptosis dysregulation are major obstacles that contribute to its poor clinical outcome [1]. Therapeutic approaches, therefore, remain a challenge for anticancer drug discovery. Natural compounds from the plant genus *Aspidistra* have been shown to possess anticancer properties because they contain up to one hundred types of biologically active saponins [22–24]. These properties are mainly attributed to the presence of polysaccharides and their derivatives [30, 31]. Previous studies reported that AA may have potential activities against cancers [25]. However, the anticancer activities of AA with respect to cancer cell proliferation and metastasis have not yet been elucidated. We reported here, for the first time, the potent pharmacological activities of AA from the endemic species *Aspidistra letreae* on the proliferation, invasion, and migration abilities of H460, H23 and A549 cells.

Several studies have reported that steroidal saponins exert strong tumor suppressive effects, including induction of apoptosis, and are intriguing candidates for anticancer research and development [30–32]. Since the dysregulation of cell death due to either insensitivity to cell death signals or overexpression of survival factors contributes to unresponsiveness to therapies, new compounds that target cancer cell death are of interest [33, 34]. As mentioned above, AA exhibited strong cytotoxicity against the examined cancer cells, with IC_50_ values ranging from 8.78 to 15.13 μM, which is consistent with a previous study on other human cancer cell types [25]. In addition, AA exhibits greater cytotoxicity in cancer cells than normal bronchial epithelial cells, with an approximately 2-fold selectivity index. The greater number is considered a higher selectivity for cancer cells [28]. Since Bcl-2 family proteins play important roles in the regulation of apoptosis, a type of programmed cell death, the balance of anti- and pro-apoptotic proteins determine the fate of the cells [35]. The irregular expression of proteins, including the upregulation of antiapoptotic or the downregulation of proapoptotic proteins in this family, commonly occurs in lung cancer cells [36]. The remodulation of protein expression is, therefore, a promising target for the treatment of lung cancer. It has been reported that saponin-related compounds induce lung cancer cell apoptosis by increasing the ratio of proapoptotic Bax/antiapoptotic Bcl-2 and decreasing antiapoptotic Mcl-1 [35, 37]. Similar to our findings, AA downregulated Bcl-2 and Mcl-1, facilitating cell death. Although the Bax level was not affected by this compound, the overall antiapoptotic signals were strongly suppressed, driving an imbalance in the Bcl-2 family that was favorable to the cell death response.

Notably, the high proliferation of cancer cells results from oncogenic transformation [38, 39]. Aberrant cell division expands a population of cancer cells with genetic instability that results in defective normal tissue functions. The limitation of cancer cell growth not only facilitates the efficacy of chemotherapy or surgery to remove cancer but also reduces the probability of cancer metastasis [40]. It has been reported that a steroidal saponin, timosaponin AIII, was able to suppress lung cancer cell growth in vivo [41]. However, our study demonstrated that a significant change in cell proliferation was observed only in cells treated with high doses of AA (Fig. 2) that are cytotoxic concentrations. The antiproliferative effect presented might be, at least in part, due to AA-mediated cell death, and the pharmacological efficacy of steroidal saponins relies heavily on polysaccharide substituents on the aglycone moiety [33, 34]. Therefore, the molecular mechanism of this effect of AA was not further deeply considered.

Cancer migration and invasion are essential steps for cancer metastasis [42]. The reorganization and morphological changes facilitate cell dissemination from the cancer tissue [8]. The secretion of enzymes, including the matrix metalloproteinase (MMP) family that degrade the extracellular environment, eases cancer cell invasion into nearby blood circulation or the lymphatic system [8]. Due to these aggressive behaviors of cancer and the physiological properties of secondary organs achieved by lung cancer cells, the chemotherapies currently available are less efficient against metastatic cancer [39]. New therapeutic agents, therefore, mostly focus on suppressing cancer metastasis [43]. A recent study revealed that diosgenin, a steroidal saponin, exhibited antimetastatic activity in human prostate cancer cells through the downregulation of MMP expression [44]. We also found that treatment with a low dose of AA markedly reduced the capacity of the examined cells to migrate and invade.

Akt signaling plays an important role in cancer survival, proliferation and metastasis [11]. Akt signaling is commonly deregulated in cancers; in particular, Akt is overactivated in human lung cancer [45]. Akt, a serine/threonine kinase, is phosphorylated and activated by several external stimuli through transmembrane proteins, including receptor tyrosine kinases, integrins and cytokine receptors [11]. The transmission of signals from these transmembrane proteins triggers the kinase enzymatic cascade that contributes to Akt phosphorylation by phosphoinositide-dependent kinase-1 (PDK1). pAkt governs several downstream signaling pathways related to cancer metastasis, including epithelial to mesenchymal transition (EMT), actin stress fiber reorganization and MMP expression [46, 47]. Inhibition of Akt function by either chemical inhibitors or RNA interference contributes to abrogating cell motility and invasion [46, 48], suggesting that Akt is an intriguing antimetastatic target. In addition, the regulatory role of Akt in cell proliferation has also been reported to occur through various downstream effectors. For example, Akt activation induces GSK3b/cyclinD1-mediated cell proliferation [49]. Akt signaling was shown to downregulate p27, a cyclin-dependent kinase inhibitor that induces cell cycle arrest [50]; in contrast, attenuation of Akt function conversely increases p27-suppressed cancer cell growth [51]. We found that AA significantly suppressed the pAkt levels, indicating that AA is a promising compound for further research and development.

## Conclusions

This study reported, for the first time, the anti-proliferation, anti-migration, and anti-invasion effects of AA on human non-small cell lung cancer cells. Compound AA was able to decrease cell viability in three human lung cancer cell lines, H460, H23 and A549 cells, whereas a smaller effect on cell proliferation was observed in these cell lines. Notably, AA significantly inhibited the migration and invasion of these examined cells via attenuation of the p-Akt/Akt signaling pathway, suggesting that AA may be a promising agent for antimetastatic therapy. Further relevant molecular mechanisms and an in vivo study will be conducted to support the potential uses of AA in the future.

## Supplementary Information

**Additional file 1: Fig. S1.** The original blot for Fig. 2d. **Fig. S2.** The original blot for Fig. 2e. **Fig. S3** The original blot for Fig. 2f. **Fig. S4** The original blot for Fig. 6.

## Data Availability

The data used and analyzed during the current study are available from the corresponding authors (DVH and VP) upon reasonable request.

## Abbreviations

AA: Aspiletrein A
GAPDH: Glyceraldehyde-3-phosphate dehydrogenase
SCLC: Small-cell lung carcinoma
NSCLC: Non-small cell lung carcinoma
HRESIMS: High-resolution electrospray ionization mass spectrometry
IC_50_: 50% Inhibitory concentration
MTT: 3-(4,5-Di-methylthiazol-2-yl)-2,5-diphenyltetrazoliumbromide
DMSO: Dimethyl sulfoxide
FBS: Fetal bovine serum
PBS: Phosphate-buffered saline
Akt: Protein kinase B
pAkt: Phosphorylated Akt
MMPs: Matrix metalloproteinases
