# Supplementary material for: Antitumor activities of Aspiletrein A, a steroidal saponin from Aspidistra letreae, on non-small cell lung cancer cells
Source: BMC Complement Med Ther. 2021 Mar 9;21:87. doi: 10.1186/s12906-021-03262-w (PMC7941985; doi:10.1186/s12906-021-03262-w)

Supplementary information for

Anti-tumor activities of Aspiletrein A, a steroidal saponin from *Aspidistra letreae* on non-small cell lung cancer cells

Hien Minh Nguyen[1], Hoai Thi Nguyen[2], Suthasinee Seephan[3], Hang Bich Do[1], Huy Truong Nguyen[1], Duc Viet Ho[2,]* & Varisa Pongrakhananon[4,5,]*

[1]Faculty of Pharmacy, Ton Duc Thang University, Ho Chi Minh City, Vietnam
[2]Faculty of Pharmacy, Hue University of Medicine and Pharmacy, Hue University, Hue City, Vietnam
[3]Pharmaceutical Sciences and Technology Graduate Program, Faculty of Pharmaceutical Sciences, Chulalongkorn University, Bangkok, 10330, Thailand
[4]Department of Pharmacology and Physiology, Faculty of Pharmaceutical Sciences, Chulalongkorn University, Bangkok, 10330, Thailand
[5]Preclinical Toxicity and Efficacy Assessment of Medicines and Chemicals Research Cluster, Chulalongkorn University, Bangkok, 10330, Thailand

*****Correspondence: varisa.p@pharm.chula.ac.th (VP); hvietduc@hueuni.edu.vn (DVH)

Fig. S1 The original blot for Fig 2D

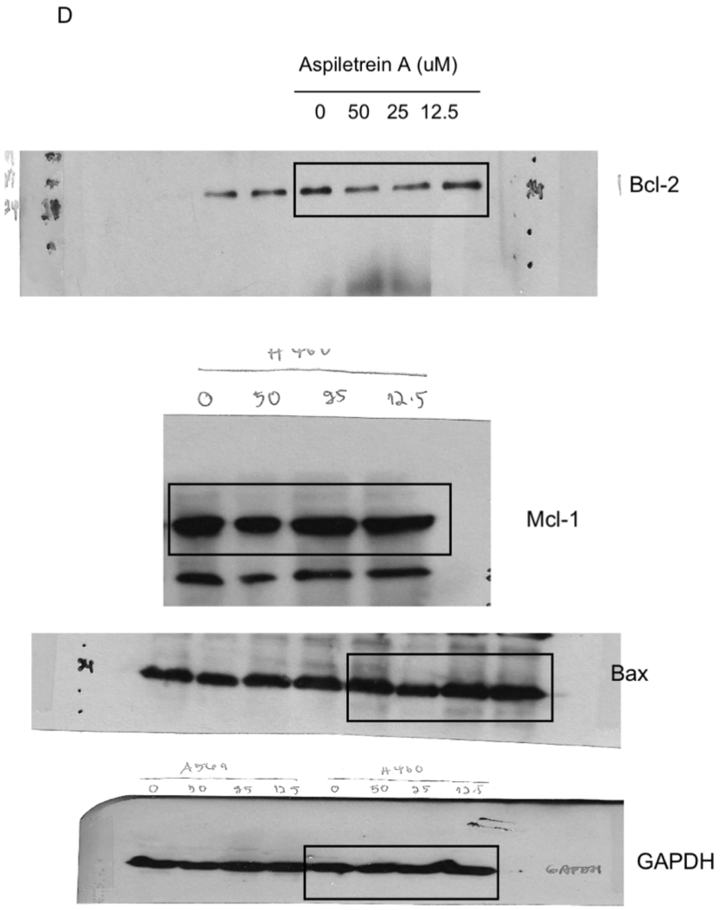

Fig. S2 The original blot for Fig 2E

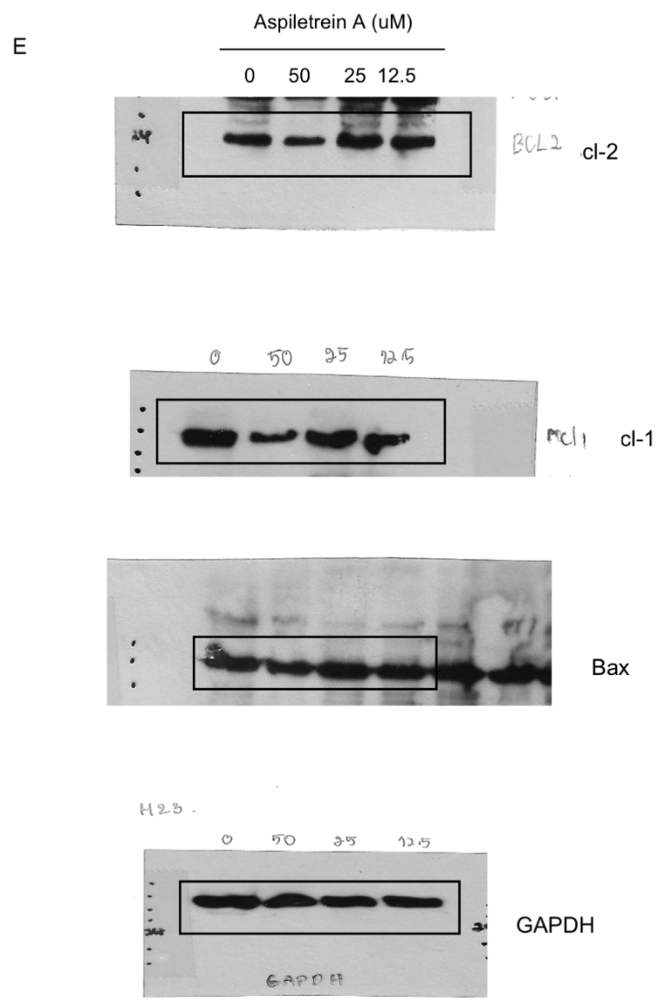

Fig. S3 The original blot for Fig 2F

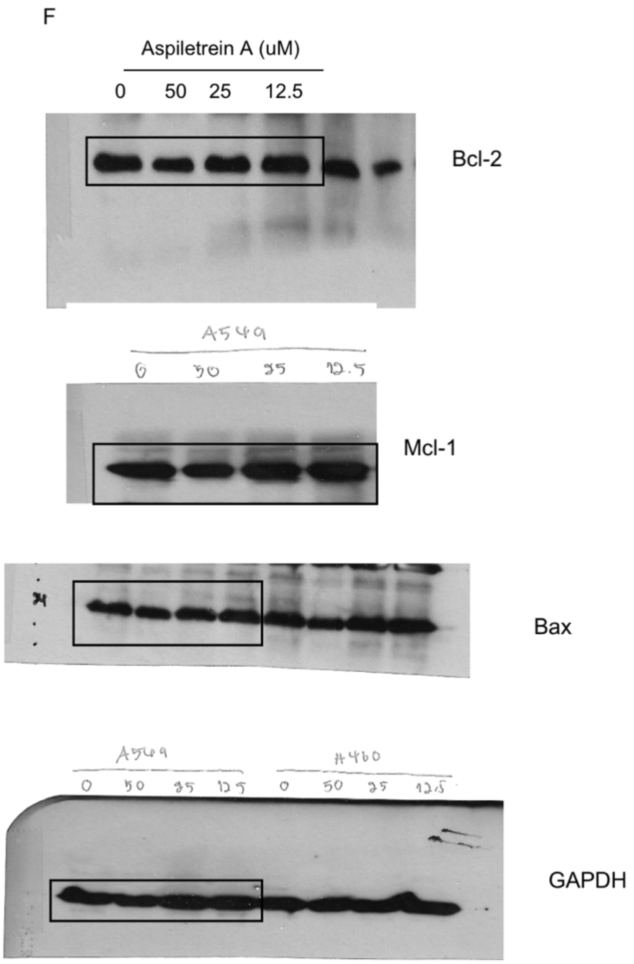

Fig. S4 The original blot for Fig 6

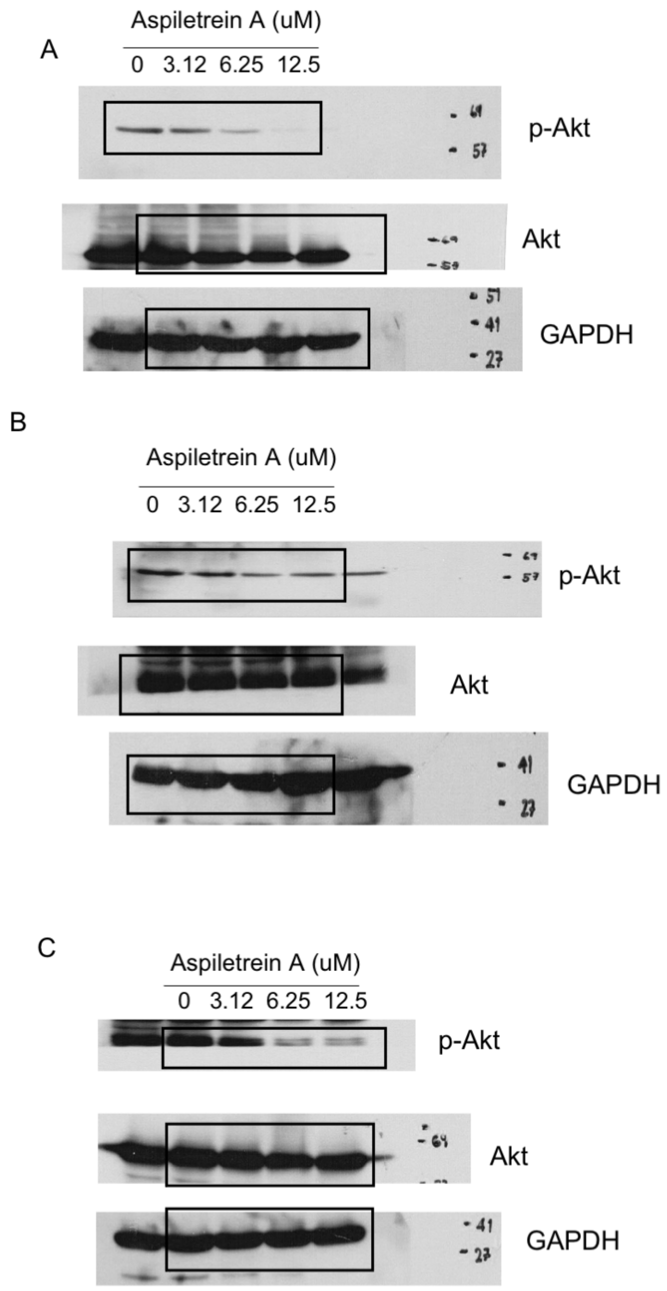

Supplement: Supplementary file 1 — Additional file 1: Fig. S1. The original blot for Fig. 2d. Fig. S2. The original blot for Fig. 2e. Fig. S3 The original blot for Fig. 2f. Fig. S4 The original blot for Fig. 6. [file 12906_2021_3262_MOESM1_ESM.pdf]
